# The School Service: What It Offers to the Newly Qualified

**Published:** 1912-09-07

**Authors:** 


### THE SCHOOL SERVICE.

### What it Offers to the Newly Qualified.

During recent years there has undoubtedly been an increase in the number of "Services" that compete for the attention of newly qualified medical men. A decade ago the choice that lay before the student who has just registered and received his diplomas was not a very wide one. He could be a consultant or a general practitioner, or he could join the Services. Then the Services meant the Army and Navy and the Indian Medical Services. To obtain a position as a medical officer of health to a town, district, or county was to achieve success which rarely came to the newly qualified. Indeed, when we exclude the consultant branch it is easy to see that then, a decade ago, there was not over much inducement held out to the student to specialise. What need was there for a general practitioner or for one destined to serve in the Army, in India, or in the Navy to take the time and money wasting D.P.H.! What need to consume so many months in attendance at the special departments or at a children's, an ophthalmic, or a throat and ear hospital! Sufficient already was the evil of a five years' curriculum that prescribed an altogether unnecessarily long list of extras which (from the point of view of the short-sighted medical student) is so much useless lumber to be discarded as soon as he has satisfied the examiners. To-day we have advanced considerably beyond the limits which were then considered finite. There is talk even of a general National Service, rumour that the tendency, both for the public and for the profession, is to urge the nationalisation of the profession and the creation of a huge establishment of rate-paid doctors.

### The New Services of To-Day.

What is very much in the glare, on the other hand, is the fact that the choice of selection has been largely increased for the newly qualified so far as the Services are concerned. We have added several new Services to the list. The medical officers of health have united and their work has become so specialised that it is not unfair to speak of a definite Public Health Service. This, again, must be held distinct from Auxiliary Services that to some extent seem to overlay it, such as the Poor-Law Service, which holds out special attractions to those who are confident that our infirmaries may yet be developed into the best hospitals in the country, and the Prison Service, which will appeal to those who are interested in criminology. Then there is, in addition, the Metropolitan Asylums Board Service for those who delight in the study of fevers; the ordinary Asylum Service for those who are interested in questions of sanity and psychological medicine; and, finally, though by no means the least of all these, the School Service. All these are new in the sense that they have been extensively developed during the last ten years. All of them claim attention from the newly qualified student who has decided that he will not enter either the ranks of the consultants nor those of the general practitioners, but will devote himself to work which, if it is badly paid, is at least regular and, on the whole, easy and devoid of that heavy responsibility which clings to private practice.

The School Service is particularly attractive to those who think the Services in general preferable to general work. Whether Service work is better than private or consulting work is, of course, a matter which each must decide for himself. We take it for granted that the newly qualified medical man is asking, "How can I become a school doctor?"

### School Inspection a Special Subject.

It is becoming increasingly recognised that school medical inspection is a very special kind of work that cannot be delegated to every registered practitioner. To be a successful school doctor demands qualities which, while they cannot honestly be styled exceptional, are, nevertheless, not indispensable in general practice. In other words, a man may be a very good general practitioner and may turn out to be a poor school doctor. Probably the reverse is equally true. The school doctor needs some capacity for organisation, tact, and special training. These necessities are now generally admitted, and he who wishes to apply for a post as school doctor should bear in mind that they are necessities recognised by the layman just as much as they are likely to be recognised by professional colleagues. What is more, the layman (in whose hands, for the most part, the appointment of the school doctor usually rests) very highly respects certain additional qualifications. The Diploma of Public Health is in his mind an indispensable possession for the candidate. It is, of course, by no means necessary that a school doctor should be able to analyse water or perform the duties of a medical officer of health, but the cachet of D.P.H. impresses the layman, and it is well to bear that fact in mind. The additional training so acquired will serve him well later on, and the year required for the diploma may be utilised for special work in children's hospitals or the children's departments of a general hospital. Such special work is indispensable. The school doctor should at least serve an apprenticeship as "clinical assistant" at a recognised children's hospital. There alone can he learn the niceties of the handling and inspection of sick children and become sensible of the vast differences between child patients and adult patients.

We should strongly advise him to devote some months to assisting or attending at certain special departments, particularly the ophthalmic, the skin, the ear and throat, and the orthopædic departments. A knowledge of eye testing and "refractions" will be highly useful to him in his school work. Needless to say, he must be well up in his general knowledge, and it is advisable that he should know something of the social conditions of the borough or town where he intends to work. With determination, some enthusiasm, and a liking for the Service he has chosen, it will not be difficult for him to satisfy these various requirements. It is true the final test can only be applied when the appointment has been accepted and when he is actually at work as school doctor. It is at present, unfortunately, impossible for the aspiring school doctor to serve as an apprentice, and all that he can do is to go to certain schools with a friend and watch the work. That is certainly advisable.

### The Pay and Hours of School Work.

On the whole the School Service offers particular attractions to the newly qualified man who has spent some time in additionally equipping himself for the work that he will be called upon to do when he enters the Service. The hours are fixed. Taking all clerical work into consideration, it is generally found that the average whole-time school doctor rarely works more than six to seven hours per day. His week-ends, from Friday afternoon to early Monday morning, are free, and he has definite holidays, ranging from a few days to several weeks in summer. His pay on the whole is fair. The London County Council pays its full-time school doctors £400 per year and its quarter-time doctors £120 per year. Country school doctors, who are generally styled "assistants to the medical officer of health," and who may be (and often are) called upon to do public health work, are paid much less, £250 being the average for a whole-time official. There is reason to believe that these salaries will be increased in the near future. A wage of less than £350 for a whole-time school doctor is unfair to the doctor and to the schools alike. Taking everything into consideration, however, it is evident that the school doctors are better paid than are medical officers of health, who have far greater responsibilities, and infinitely better than ships' surgeons or hospital residents, whose energies are fully utilised by their employers, and whose work is in general of a much more exacting nature. Add to this the fact that the duties of the school doctor are generally easy and always interesting (to those at least who are interested in children), and it will be seen that the attractions offered by the Service compare favourably with those put forward in other quarters.

On the other side must be placed the fact that the work is monotonous. It is largely routine work, which may, unless the doctor is careful and enthusiastic, degenerate into slovenliness. The school doctor is concerned only with the diagnosis of defects; he does not deal with the treatment of lesions found. Unless, therefore, he is keen and keeps himself up to date in regard to treatment and general medicine, there is a danger that he might get stereotyped and in close attention to his specialty lose that general interest which every doctor should encourage in medicine. That danger is not, of course, confined to school doctors or to medical inspection of school children; it attaches to every specialty that is to a certain extent exclusive, and the only effectual safeguard against it is enthusiasm and the decision not to let pediatrics usurp all the energy and attention that the doctor can devote to his profession. The monotony of routine work may be galling to some; certainly it can never prove unbearable to the school doctor who is actively interested in his duties, for there is a constant change of interest, and the field for exploitation is large. To cultivate that field wholly and most successfully we counsel the school doctor to interest himself in the child-life in his special school district. Let him make friends with individual members of the Care Committees and School Managers; let him visit Mrs. "Ward's" play centres and actively interest himself in the work that is being carried on under the central supervision of the Passmore Edwards' Branch. Let him attach himself to some children's club. Extra work all this, but work that will pay—pay in interest, in pleasure, and, above all, in the satisfaction of working to benefit some one.

The school doctor's daily routine duties vary in different parts of the country. Generally he has the benefit of the help of an experienced school nurse, who, if she is capable, relieves him of much of the drudgery attendant upon school inspections and considerably lightens his work. She arrives at the school some time before the doctor is due and makes all preliminary arrangements, but the doctor has the responsibility and on him devolves the duty of explaining defects to parents or teachers.

### London County Council Requirements.

The London County Council School Service, which, under Dr. Kerr's able supervision, has become a model for the country, requires the doctors to "make systematic visits at the appointed times to the schools allotted to them, paying due regard in all such work to the necessity of using as much tact as possible with teachers, parents, or children, and of effecting as little disturbance of school routine as necessity will allow . . . and to report upon the environment, structure, and condition of school buildings, furniture, ventilation and heating, school organisation, educational methods and apparatus, physical condition of the children, and any other matters coming under their notice in so far as they affect, or are likely to affect, the health of teachers or children and the physical development of the latter . . . to confer with, advise, and act in conjunction with head teachers regarding matters of hygiene and health . . . to examine children in the schools pursuant to the provisions of the Elementary Education (Administrative Provisions) Act, 1907 . . . to take part in special researches upon the conditions of healthy school life." Visits are made between the hours of 9.30 and 12.30 in the forenoon and between 2.15 and 4.30 in the afternoon. During a session the doctor is supposed to inspect from twenty to thirty children; with a little practice the maximum number can easily be inspected. Now and then when a child presents a special lesion the inspection, to be thorough, may take an hour or more; in these cases it is, of course, advisable to set the case aside for examination at a later time, such cases being regarded as "specials."

With method the school doctor will find that the sessional examinations are by no means wearying. If he is a student of children every session may be made instructive and interesting in various ways. Those who wish to devote their attention to research work will find the School Service offers exceptional opportunities for carrying out investigations on original lines. The results from such investigations are bound to be more conclusive and valuable than those emanating from the out-patient departments of hospitals, since the material available for the school doctor is so much more varied and so much greater.

Opportunities for Research and a Warning.

There are a host of subjects that clamour for special research and investigation. Such commonplace lesions as enlarged glands, caries of the teeth, and flat-feet offer scope for the energies of the enthusiastic school doctor, while it is evident that anyone who studies nervous diseases and their incidence in school children will find much that will be of use and value to the profession at large. The subjects of school buildings, school bacteriology, psychiatry, and hygiene generally will need special apostles, and the school doctor will find himself in a position to do his share in the way of special research. He should join the School Medical Officers' Association, the subscription to which is merely half a guinea. The meetings of the Association are always interesting, and the value of the Society is undoubted and will increase in course of time. Membership of the Child Study Society is also advisable, if merely for the sake of the well-selected library available to members. It cannot honestly be said that the average school doctor is likely to learn much from the desultory debates which are carried on by this Society. In addition the school doctor should join some good Medical Society and keep up his interest in general medicine and the progress made in departments which lie outside his speciality. Needless to say, he should also attach himself to a Medical Defence Union or Association. On more than one occasion his work carries him into dangers which, however easily they are usually circumvented, may possibly call for the interference of the Union's solicitor. In any case the annual subscription paid to such a Defence Union is one of the best investments that the medical man, whether school doctor or not, can make.

In order to become a school doctor—for the original question has not yet been answered—the aspirant should aim at acquiring a good general knowledge of medicine and a special knowledge of pediatrics. In addition he should study hygiene and sanitation, bacteriology, diseases of the skin, ear, nose and eye, and deformities. The taking of the D.P.H. is certainly advisable, but there is no reason why he should not apply for a vacancy even before he has gained that diploma. All that is necessary is then to study the advertisement columns. When a vacancy occurs the candidate should forward his application, together with testimonials.

It is well to lay stress on the advisability of having special testimonials which emphasise the candidate's fitness for the School Service by detailing his qualifications in one or two special branches. There is already very keen competition among candidates for the Service (for twenty vacancies on the London County Council last month no fewer than 380 applications were received, and for a badly paid assistancy in a country district there were recently seventy-five applicants), and the candidate should not neglect any legitimate opportunity of impressing the selection committee with his fitness and with his keenness. Once the post has been secured strict attention to the duties will show the Care Committees and the authorities generally that the school doctor is a treasure that cannot very well be spared—a matter of some importance when it comes to the readjustment of salaries.

